# Model of driving factors for success in public health project management using structural equation modeling

**DOI:** 10.1038/s41598-024-75437-7

**Published:** 2024-10-20

**Authors:** Carolina Santos, João Varajão, Nilton Takagi, A. Manuela Gonçalves

**Affiliations:** 1 NOVA National School of Public Health, Public Health Research Centre, Comprehensive Health Research Center, NOVA University, Lisbon, Portugal; 2 ALGORITMI Center/LASI, University of Minho, Guimarães, Portugal; 3 Institute of Computing, Federal University of Mato Grosso, Cuiabá, MT Brazil; 4 Centre of Mathematics, University of Minho, Guimarães, Portugal

**Keywords:** Public health, Success factors, Success criteria, Project success, Project management, Health services, Public health

## Abstract

In a context where pandemic crises and chronic conditions are a constant and increasing threat, the success of public health projects is absolutely critical. However, little is known about the factors that influence the success of projects that aim to provide conditions for people to be healthy and prolong the life of the population as a whole. A mixed-method study was carried out to fill the literature gap, resulting in a new model of success factors for public health projects. The research work theorizes the success factors that impact public health project success, providing relevant knowledge for project managers and contributing to the successful management of public health projects.

**Supplementary Information:**

The online version contains supplementary material available at 10.1038/s41598-024-75437-7.

## Introduction

The goals of public health have expanded over the last decades, and the results of public health initiatives are now reflected in the decrease of thousands of worldwide cases of measles, diphtheria, and polio^1^, to name a few. Furthermore, according to Turnock^1^, public health focuses on prevention, such as the use of seat belts to reduce the number of deaths from accidents or even protection policies for the blood supply system to avoid infections of both hepatitis B and C and human immunodeficiency virus (HIV), whose treatment would cost billions of dollars.

Public health initiatives fight infectious diseases that are difficult to address without collective action^2^. For instance, the COVID-19 pandemic has prompted public health agencies worldwide to carry out initiatives to issue guidelines regarding prevention measures—such as washing hands regularly, covering mouth and nose, avoiding contact with people who have the symptoms of the disease, avoiding traveling to cities and areas affected by the pandemic, etc.—and national recommendations in the case of identified contagion^3^. The enormous stress caused by the COVID-19 pandemic has shown vulnerabilities and weaknesses in the public health and healthcare systems^4,5^.

Public health goals are achieved through projects^6^. A project is a temporary organization to which resources are assigned to deliver beneficial change. It is a useful way to introduce innovations, address new needs, or find solutions to problems that the status quo does not accommodate^7^. According to the World Health Organization^8^, public health projects are initiatives aimed at improving the health and well-being of populations. These projects focus on preventing disease, prolonging life, and promoting health through organized efforts and informed choices of society, organizations, public and private sectors, communities, and individuals. These projects can vary widely in scope and focus. Still, they generally involve activities such as^9^: disease prevention and control (implementing vaccination programs, conducting health screenings, and promoting hygiene practices to prevent the spread of infectious diseases); health education and promotion (educating the public about healthy lifestyles, nutrition, exercise, and preventive health measures to reduce the risk of chronic diseases); environmental health (addressing environmental factors that affect health, such as air and water quality, waste management, and pollution control); health policy and advocacy (developing and advocating for policies that promote health equity, access to healthcare, and the reduction of health disparities; emergency preparedness and response (preparing for and responding to public health emergencies, such as natural disasters, pandemics, and bioterrorism).

Public health projects are essential for protecting and improving the health of entire populations, from local communities to global regions. However, there has been some criticism regarding these projects^10^: absence of clear goals, lack of evidence-based interventions, low-quality evaluation criteria, and poor reporting on successes and failures. Recognizing success factors is essential to avoid the failure of these projects. Ika^11^ defines success factors as conditions, events, and circumstances contributing to project results, i.e., they are variables that contribute to the likelihood of success^12^. If these factors are not identified, monitored, and controlled, they can jeopardize an endeavor^13^. When properly considered, success factors reduce the uncertainties inherent in project development and contribute to improved results^14^.

For the good of public and private institutions, as well as for society as a whole, public health projects and programs must succeed. The success of a public health project mainly depends on its global impact on the target population^15^. There is a body of knowledge focused on project success factors that are presented in an extensive number of papers in the literature^16–22^. However, in the case of public health projects, studies are practically non-existent. Furthermore, the extant knowledge is not sufficient to assess whether the “classical lists” of success factors fit into public health projects focused on the health and wellness of groups and populations since these projects have specific aspects^23^. Additionally, studies focusing on project success factors typically identify them but do not focus on their particular contribution to project success.

Our study contributes to filling the knowledge gap by proposing an empirically validated theoretical model of success factors for public health projects. We carried out a mixed-method study that included both an exploratory and a confirmatory analysis. The results enabled us to identify success factors and link them to observed project success. This work theorizes the success factors that impact the overall success of project management and public health projects. When integrated with Success Management practices^24–27^, this work provides support for the successful management of public health projects.

The paper employs the following structure. In the next section, we present the background regarding public health projects, success factors, and success criteria. In the third section, we describe the research method and the theoretical model of success factors. In the fourth section, we present the results of descriptive and inferential statistics. In the fifth section, we discuss the study results. Finally, we present implications for theory and practice, as well as the limitations that can be addressed in future research.

## Background

### Public health projects

Population-based public health projects are focused on the determinants of health for defined populations and are concerned with providing conditions for individuals, groups, and society as a whole to be healthy^28^. It is important to improve governance in healthcare, for example, by assessing value generation^29^. Interventions regarding populations’ health integrate the “art and science” of preventing disease, prolonging life, promoting physical and mental health, sanitation, personal hygiene, control of infectious diseases, and organization of health services^30^. Public health projects can assist in decision-making at the local, national, and international levels in areas such as environmental and occupational health policies, injury prevention policies, and nutrition and food safety policies^31^. The types of projects in the scope of public health are guided by predefined objectives, as follows^10^:

- **Research projects**: The main goal of this type of project is to improve the decision-making process by increasing knowledge through the development of the “evidence base”. Part of these projects is related to identifying health problems in a given population and the factors that are contributing to such problems. Another part is related to the evaluation of the quality and effectiveness of a public health intervention project by analyzing the implementation process and both the short and long-term results;
- **Development projects**: This type of project involves the development and pre-testing of a public health intervention to address a specific problem in a given population or target group. The projects are focused on a detailed analysis of the problems, which results in the selection of relevant objectives and intervention strategies with demonstrated or expected effectiveness;
- **Implementation projects**: This type of project is focused on the wider dissemination and implementation of an existing public health intervention in a particular target group or population. These projects should feature a careful analysis of both the target audience and implementation conditions and usually require the involvement of third parties (intermediaries) familiar with the target population and the local context to support the implementation process. A specific form of this type of project is community projects, which follow a bottom-up approach and strongly emphasize the participation of community stakeholders.

Projects in public health can be a combination of more than one type of project. Oftentimes, these combined projects feature subprojects with specific objectives and expected outcomes.

Some examples of public health projects are as follows: the use of artificial intelligence applications and telehealth as solutions for protecting public health in pandemic times^32^; analyses of how perceptions about vaccines and anti-vaccination movements impact public health^33,34^; definition of public health measures to assess attitudes and behaviors to reduce transmission of COVID-19^35^. Knowing the variables that impact project success is a critical requirement to effectively manage health projects and thus increase the likelihood of positive outcomes for the citizens.

### Project success factors

Many projects fail or do not fulfill their goals^36,37^. One of the reasons for such project results is related to success factors^38^, which constitute a set of circumstances, facts, or influences that contribute to the project outcomes^39^.

Success factors have been intensively explored by project management research over the past three decades^14,40–43^ and are a critical element of Success Management^25,26^.

Some of these success factors have already been associated with project management in the public health sector. Research conducted on maternal, newborn, and child health^44^ has identified factors that directly impact project success: for example, unrealistic planning (planning factor), inadequate working environment (stakeholders’ management, mission, and environment, leadership factors), political interference (organizational culture factor), and inefficient knowledge acquisition (project execution and control, monitoring and evaluation factors). Milat et al.^45^ assessed success factors by focusing on scaling up public health actions in low and middle-income country contexts. Among the key success factors found were the following: the importance of establishing monitoring and evaluation systems; economic and cost modeling of intervention approaches; active involvement of a variety of implementers and the target community; tailoring the scaled-up approach to local context; use of participatory approaches; systematic use of evidence; infrastructure to support implementation; strong leadership and advocates; political will; well-defined scale-up strategy; and strong advocacy.

### Project success criteria

Understanding and evaluating the success of projects is crucial^46^. Project success is measured against project objectives and success criteria^47–49^. The success criteria form the set of principles or standards through which a judgment of project success is made^26,50^. They are the informal and formal measures by which project goals and impact on stakeholders are assessed^51,52^.

The classical success criteria are related to scope, time, costs, quality, and goals: delivering the product, finishing the project on time and within budget, and achieving the project’s short, medium, and long-term goals^38,53–56^.

In more recent times, other criteria than time, cost, and quality are considered in project success evaluation^52,57–69^. One of such criteria is related to stakeholder satisfaction (e.g., end users)^70^. Some examples are: Satisfaction with the final product—the final product meets requirements and specifications defined by the project owner^50^; Satisfaction and benefits for the client—the project owner is satisfied with the results, and the planned benefits are generated^38,43,54,68,71^; Satisfaction and benefits for stakeholders—stakeholders are satisfied during project implementation, and at project closure the planned benefits and outcomes are generated to the network of stakeholders^38,43,54,68,71^.

The overall project success is made up mainly of two different elements: the success of project management and the success of the project product^54^. Successful project management, on the one hand, depends on the management process, namely on the project’s successful completion in relation to the three dimensions mentioned above of scope, time, and cost, which reveals the extent of its efficacy and efficiency. Product success, on the other hand, is primarily concerned with the effects of the project’s outputs (products or services) in the post-project phase. Cooke-Davies^72^ noted that ensuring project deliverables success is more difficult than ensuring project management success.

## Method and theoretical model

Our research followed a mixed approach composed of qualitative and quantitative methods. Mixed methods strategies provide a powerful mechanism for researchers to address research situations and make contributions to both theory and practice^73^. After a narrative literature review, our research started with a qualitative study (exploratory phase) to define the theoretical model, followed by a quantitative study (confirmatory phase) to corroborate the model.

### Qualitative study

Prior to the qualitative study, a narrative literature review was conducted to find evidence concerning “generic success factors” and “public health success factors”. Over forty papers were considered eligible and subsequently analyzed. Based on this literature review, a preliminary theoretical model of success factors was developed.

To refine and evolve the preliminary model, a set of interviews was carried out with experts in both project management and public health who worked in the public, private, and social sectors. The selection of interviewees was determined according to their expertise in public health projects and project management. Among the interviewees are directors of public and private institutions, a member of the Ministry of Health, a city mayor, a health project manager, and professors who are also researchers and health project coordinators.

**Fig. 1:**
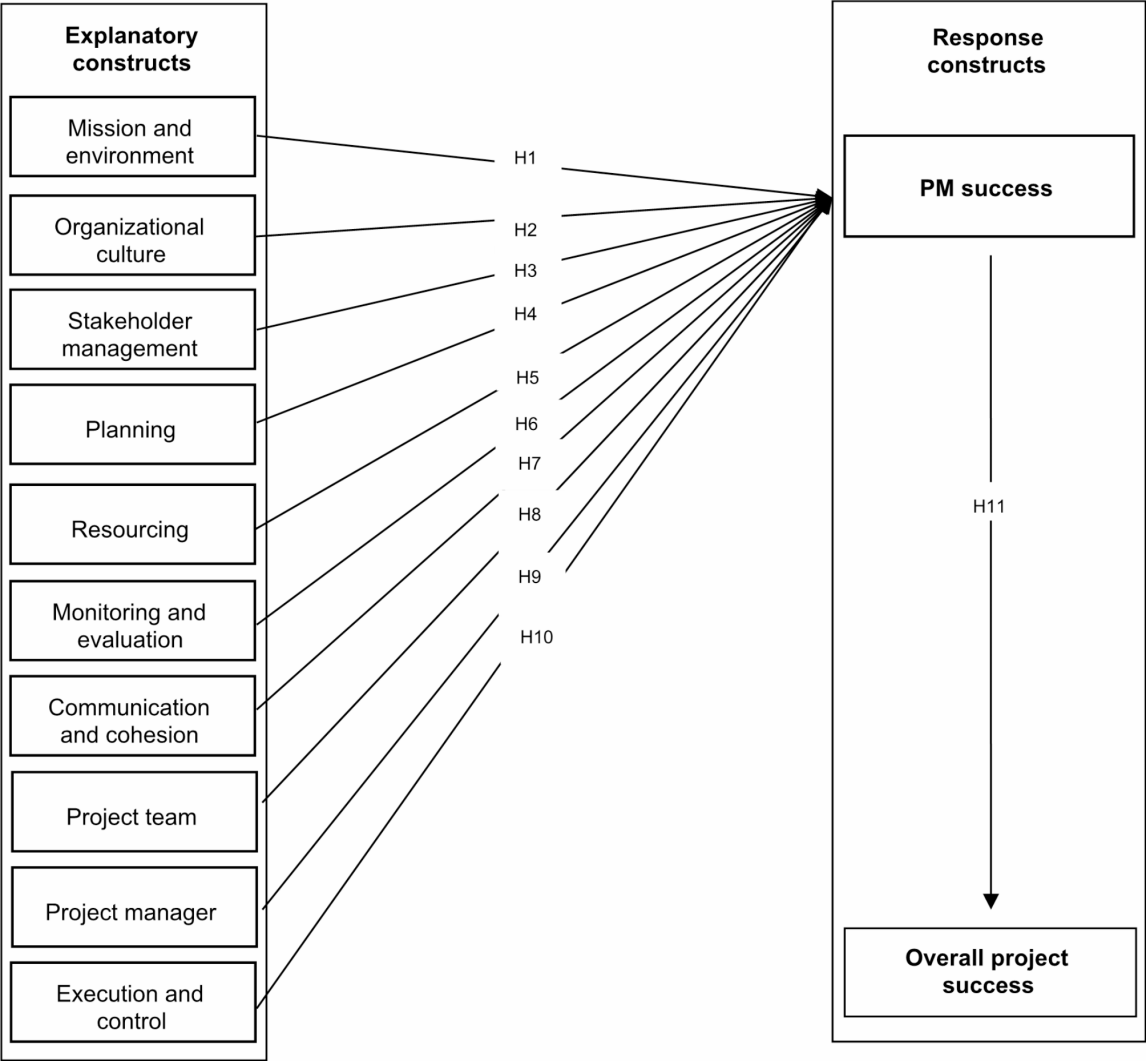
Theoretical model.

Prior to conducting the interviews, a script was developed addressing the success factors and success criteria in the scope of public health projects. The script was pre-tested with two individuals, and minor changes were made regarding wording. Nine semi-structured interviews were then conducted. The interviews were audio recorded with the respondents’ permission, with seven face-to-face interviews and two videoconference interviews. This step enabled the identification of new important themes (not present in the preliminary model) and the reformulation or removal of variables, leading to the evolution of the prior model (which was based on the literature review). The theoretical model (Fig. 1) presented in “Theoretical model and measurement” is the final result of the qualitative study.

### Theoretical model and measurement

Achieving success in public health projects is critical for any public institution since it directly impacts the lives of citizens. Figure 1 presents the theoretical model of success factors resulting from the qualitative study. Table 5, in the appendix, presents details of the constructs. Each arrow in the figure represents the hypotheses to be tested.

Mission and environment consists of factors related to the project’s *raison d’être* and respective context. The project’s purpose can impact its success. The purpose must be in line with the public environment and be clear, realistic, and achievable. These success factors are associated, for example, with the public interest in the project’s results and its contribution both to priority public health programs and promoting the organization’s strategic goals^42,43,71,74–79^. Thus, we propose the following hypothesis:

#### H1

There is a statistically significant relationship between the construct “mission and environment” and the success of project management (PM success).

**Organizational culture** is a set of values, beliefs, and behavioral norms that guide how the organization’s employees perform their work^80,81^. In the public sector, for example, initiatives that promote less bureaucracy and more flexibility can be factors directly linked to project results. These organizational factors can be related to organizational structure, work environment, and knowledge sharing^41,74–77,82–84^. Thus, we establish the following hypothesis:

#### H2

There is a statistically significant relationship between the construct “organizational culture” and the success of project management (PM success).

S**takeholder management** consists in identifying, analyzing, and proactively engaging stakeholders to achieve goals^85^. In public health projects, it is important to map strategic partners and affected communities. The related success factors may be associated, for example, with involvement, trust, confidence, compatible development priorities, community engagement, end-user involvement, and communication and active listening of all stakeholders^12,41,43,63,65,71,74–78,82,84,86,87^. Thus, we put forward the following hypothesis:

#### H3

There is a statistically significant relationship between the construct “stakeholder management” and the success of project management (PM success).

**Planning** consists generally in the definition of tasks, resources, and other actions necessary to be performed to achieve the proposed objectives^88^. These success factors are related, for example, to the quality (accuracy and consistency) of project planning, clear identification of success criteria and success factors, risk identification and response plans, and detailing of scope and timelines^41,63,75,76,78,89–91^. Thus, we formulate the following hypothesis:

#### H4

There is a statistically significant relationship between the construct “planning” and the success of project management (PM success).

**Resourcing** can include supplies, materials, equipment, services, and team members^92^. In project management, resources must be managed accordingly to ensure that they are sufficient to successfully complete the project^93^. Its respective success factors are accurate budgeting, suitable funding to support the project plan and assure project completion, and allocation of sufficient resources when needed^59,74–79,84,87^. Thus, we advance the following hypothesis:

#### H5

There is a statistically significant relationship between the construct “resourcing” and the success of project management (PM success).

**Monitoring and evaluation** consists in tracking, reviewing, and reporting on project progress to evaluate whether the planned actions are being performed as expected and whether objectives are being accomplished^94^. Monitoring and evaluation can result in preventive or corrective actions to keep the project on track for success^26^. These success factors are related, for example, to the quality of the project management information system and appropriate performance indicators^43,63,74,76^. Thus, we propose the following hypothesis:

#### H6

There is a statistically significant relationship between the construct “monitoring and evaluation” and the success of project management (PM success).

**Communication and cohesion** is the process of acquiring all relevant information, interpreting it, and effectively disseminating it to the people who may need it^95^. Communication is one of the areas that impact project success the most^96^. Its respective success factors are appropriate information available for all key project stakeholders, suitable communication channels and information flows, cooperation, cohesion, trust, and interpersonal relationships within the project team^41,43,63,74–76,82,84,87,97^. Thus, we establish the following hypothesis:

#### H7

There is a statistically significant relationship between the construct “communication and cohesion” and the success of project management (PM success).

**Project team** consists of a group of people with complementary skills, from different disciplines and/or functional areas, who become a team with the objective of completing a project^98^. The project team can impact project success through factors such as motivation, experience, and technical competencies to carry out the work^41,43,63,66,71,74–76,78,99,100^. Thus, we propose the following hypothesis:

#### H8

There is a statistically significant relationship between the construct “project team” and the success of project management (PM success).

The **project manager** is the person assigned by the performing organization to lead the project team and is responsible for achieving the project’s success^101^. Project manager success factors are related to conflict-solving skills, competency, background in project management, leadership skills, ability to delegate authority, good perception of his role and responsibilities, commitment to the project, and experience in PM^43,63,71,74,76,89^. Thus, we put forward the following hypothesis:

#### H9

There is a statistically significant relationship between the construct “project manager” and the success of project management (PM success).

**Project execution and control** consists of processes and activities performed to complete the work defined in the project management plan in order to meet project requirements^92^. These success factors are related to the use of project management standards (methods, tools, techniques), streamlining of processes, troubleshooting, ability to deal with unexpected crises and deviations from the plan, ability to make a “fresh start” when mistakes are identified^42,43,59,63,74,76,77,82,84,89,97,99^. Thus, we advance the following hypothesis:

#### H10

There is a statistically significant relationship between the construct “execution and control” and the success of project management (PM success).

**Project Management Success (PM Success)** refers to how efficiently a project achieves its goals and objectives^20^. The following are among the most commonly used success criteria in the literature related to project management: meeting the schedule, meeting the budget, achieving project objectives, and stakeholder satisfaction^42,59,76,82,84,89,102^. Another important dimension of project success relates to the success of project outputs^25^. On the one hand, the success of the products and services and the success of project management are independent, but a project management failure might compromise the success of the outputs. As a result, it is important to note that the project and any final products or services should not be viewed in isolation^103^. On the other hand, the link between project management and overall project performance, which is hard to measure and model, remains somewhat unexplored, as it usually involves complex constructs^43,104^. Therefore, we propose the following hypothesis:

#### H11

There is a statistically significant relationship between the construct “success of project management” (PM success) and overall project success.

### Quantitative study

We carried out a questionnaire-based survey to test the theoretical model. The survey involved project managers and team members of public health projects (see Table 1) that contributed to the execution of the Priority Public Health Programs, co-financed by International Programs (Third EU Health Program and EEA Grants) and by the Gilead Genese Program^®^. Other projects were also identified, considering the Portuguese Good Health Practices Award.

The questionnaire included open and closed questions and was organized into the following three sections: (1) Section 1 – descriptive data regarding the respondent and their organization; (2) Section 2 – characterization of the project (e.g., schedule, budget, sources of funding, partnerships, type of public health intervention), and relevant data to measure the achieved success; (3) Section 3 – data concerning the measurement of the success factors constructs. The variables of the explanatory and responsive constructs were measured using ordinal scales. The measuring variables are detailed in the Appendix.

**Table 1:** Number of projects invited for participation (by program).

Program description	Invited projects (*n*)
EEA Grants	21
Third EU Health Program	12
Priority Health Programs Prevention and Tobacco Control Healthy Food Promotion Physical Activity Promotion Diabetes Cardiovascular-brain diseases Respiratory diseases HIV/AIDS and Tuberculosis Infection Prevention and Control of Infections and Antimicrobial Resistance Oncological diseases	49
Gilead Genese Program	8
Portuguese Good Health Practices Award	16

The survey was piloted by conducting three pre-tests to identify potential ambiguities and exclude any questions that could lead to misinterpretation. Some adjustments regarding wording were made based on this feedback. The link to the questionnaire (created in Google Forms) was submitted by email after a telephone call to the project manager based on the following objectives: (1) identifying whether the project met the eligible criteria (completed less than 24 months ago); (2) explaining the goals of the research; (3) ensuring anonymity and relevance of participation; (4) maximizing the chances of response. We also requested project managers to forward the questionnaire link to their project team members. The email with the invitation to participate in the study was sent to 106 project managers, and a follow-up telephone contact was then made (15 to 30 days after sending the email) to maximize the response rate.

A total of 142 responses were received: 85 from project managers and the remaining from team members. The global response rate cannot be accurately calculated, as the link for the questionnaire was sent by project managers to an unknown number of project team members. The data were analyzed using statistical analysis software (*IBM SPSS Statistic 24* and *AMOS*). The quantitative analysis enabled us to find statistically significant relations between the factors and the degree of success achieved by projects. The literature supports that quantitative analysis is the most suitable for finding incidence, distribution, and relations between variables in a natural context without manipulation^105^.

## Results

### Reliability

The internal reliability of the model was investigated using Cronbach’s Alpha. The initial model comprised 86 variables. After confirming the factorial analysis, five explanatory variables and one responsive variable were removed (see Appendix for details). The alpha coefficient for all the constructs, except “monitoring and evaluation”, was above the acceptable threshold level of 0.7^106^. An alpha coefficient value above 0.6 is considered acceptable in social science research^107,108^.

### Frequency and descriptive statistics

Table 2 presents the sample demographics.

**Table 2:** Sample demographics.

Gender	*N*	%	Project role	*N*	%
Male	33	23.2%	Project manager	85	59.9%
Female	109	76.8%	Team member	57	40.1%

Education	N	%	Age (year)	N	%
Lower than a bachelor’s degree	4	2.8%	< 35	31	21.8%
Bachelor	73	51.4%	35–45	50	35.2%
Master’s degree	41	28.9%	46–55	48	33.8%
Doctorate’s degree	24	16.9%	> 55	13	9.2%
			Average	43	

Project Management Experience	N	%	PMO	N	%
Yes	82	57.7%	Yes	44	31.0%
No	60	42.3%	No	98	69.0%

PM software	N	%	Project budget (Euro)	N	%
Yes	9	6.3%	< 1000	23	16.2%
No/Do not know	133	93.7%	1000–59,999	16	11.3%
			60,000–500,000	27	19.0%
			> 500,000	14	9.8%
			Do not know/no answer	62	43.7%

Project timeline (month)	N	%	Project funding	N	%
< 12	57	40.1%	Organization budget	67	37.2%
12–23	15	10.6%	Community funds	21	11.7%
24–35	54	38.0%	EU grants	36	20.0%
> 35	13	9.2%	Sponsors	18	10.0%
Do not know/no answer	3	2.1%	Other	20	11.1%
Average	24		Do not know/no answer	18	10.0%

The sample consisted of 142 responses, all fully completed and considered valid. The majority of respondents were project managers (59.9%), female (76.8%), aged 43 on average, holding a master’s degree (28.9%), and having previous experience in PM (57.7%). The majority (69.0%) answered that there is no project management office (PMO) and that no PM software (93.7%) is used in the organization. The project timelines averaged 24 months, and the budget was €400.938, mainly obtained from the promoting organization’s own budget (37.2%) and EU grants (20.0%).

### Inferential statistics: correlations and modeling

#### Spearman’s correlations

The strength of the association between success factors and success criteria was measured by bivariate analysis, and a non-parametric Spearman’s correlation was applied. The averages, standard deviations (SD), and Spearman’s correlation matrix of the constructs are presented in Table 3. We observe that there is a statistically significant positive relationship between all the independent variables and the success constructs.

**Table 3:** Averages, standard deviations, and Spearman’s correlations of the latent variables.

	Average (SD)	1	2	3	4	5
Mission and environment	5.781 (0.816)					
Organizational culture	5.408 (1.044)	0.406**				
Stakeholder management	5.162 (1.165)	0.593**	0.292**			
Planning	5.591 (0.920)	0.556**	0.361**	0.507**		
Resourcing	4.510 (1.678)	0.375**	0.255**	0.336**	0.501**	
Monitoring and evaluation	4.442 (1.673)	0.327**	0.245**	0.404**	0.566**	0.513**
Communication and cohesion	5.714 (1.069)	0.567**	0.347**	0.595**	0.699**	0.454**
Project team	5.750 (0.962)	0.420**	0.305**	0.421**	0.427**	0.443**
Project manager	5.843 (1.058)	0.410**	0.379**	0.361**	0.422**	0.353**
Execution and control	5.156 (1.136)	0.588**	0.588**	0.607**	0.607**	0.428**
PM success	3.414 (0.781)	0.345**	0.208**	0.405**	0.401**	0.355**
Project success	5.937 (0.893)	0.236**	0.205*	0.265**	0.370**	0.244**

	6	7	8	9	10	11
Mission and environment						
Organizational culture						
Stakeholder management						
Planning						
Resourcing						
Monitoring and evaluation						
Communication and cohesion	0.506**					
Project team	0.534**	0.563**				
Project manager	0.479**	0.535**	0.776**			
Execution and control	0.568**	0.740**	0.547**	0.517**		
PM success	0.524**	0.504**	0.482**	0.396**	0.373**	
Project success	0.225**	0.403**	0.389**	0.304**	0.317**	0.486**

* *p* < 0.05; ** *p* < 0.01.

#### Structural equation modeling

**Fig. 2:**
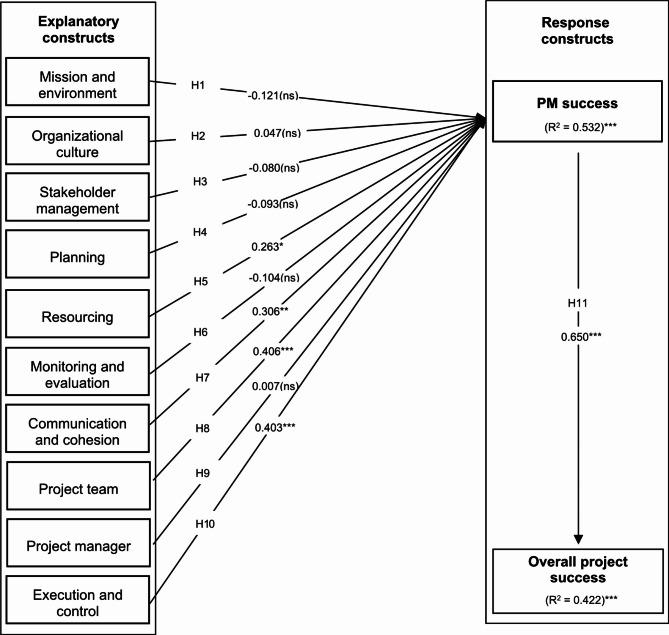
Model and regression coefficients. * *p* < 0.05; ** *p* < 0.01, *** *p* < 0.001, ns (not significative).

The model adequacy was assessed by Structural Equation Modeling (SEM). SEM is a robust technique for evaluating, modifying, and testing relationships between variables^109^. The estimation method used was the maximum likelihood method. The quality of fit was evaluated using the following indexes: χ2 statistic, Root Mean Square Error of Approximation (RMSEA), Tucker-Lewis Index (TLI), and Comparative Fit Index (CFI). All the estimates presented are standardized. The specified model presented acceptable adjustment quality indexes. The model and regression coefficients are shown in Fig. 2.

After analyzing the standardized coefficients and their degrees of significance, it is possible to confirm the following hypotheses regarding the surveyed projects:

##### H5

There is a statistically significant relationship between the construct “resourcing” and the success of project management (0.263, *p* < 0.05).

##### H7

There is a statistically significant relationship between the construct “communication and cohesion” and the success of project management (0.306, *p <* 0.01).

##### H8

There is a statistically significant relationship between the construct “project team” and the success of project management (0.406, *p* < 0.001).

##### H10

There is a statistically significant relationship between the construct “execution and control” and the success of project management (0.403, *p* < 0.001).

##### H11

There is a statistically significant relationship between the construct “success of project management” and overall project success (0.650, *p* < 0.001).

The behavior of the variables that measured the success factors happened as expected, so positive variations in the explanatory constructs benefited PM success and overall project success, and the magnitude of this relationship is more expressive regarding the constructs “project team” and “execution and control”. Overall, the explanatory constructs define a 53.2% variance in PM success. In addition, variations in PM success, measured by the achievement of goals, results, and end-users satisfaction, defined a 42.2% variance in overall project success.

#### Findings – correlation and cross-validation

The findings from the correlation and modeling analysis are presented in Table 4, which shows the standardized coefficients from SEM in ascending order, along with the correlation values from Spearman’s correlations and effect sizes based on Cohen^110^: large correlations are described as being greater than 0.50; medium correlations ranging from 0.30 to 0.49; and small correlations ranging from 0.10 to 0.29. The SEM results support the results of the correlation tests.

**Table 4:** Combined results for the correlation test and SEM.

Ref	Independent variables	Dependent variable	Spearman’s correlations	Sig.	SEM results		Effect size
					Standardized coefficients	Sig.	
H5	Resourcing	PM success	0.355	< 0.01	0.263*	< 0.05	Small
H7	Communication and cohesion	PM success	0.504	< 0.01	0.306**	< 0.01	Medium
H8	Project team	PM success	0.482	< 0.01	0.406***	< 0.001	Medium
H10	Execution and control	PM success	0.373	< 0.01	0.403***	< 0.001	Medium
H11	PM success	Overall project success	0.486	< 0.01	0.650***	< 0.001	Large

## Discussion

This research identified positive relationships between a set of success factors constructs and PM success/overall project success. The final model defines a 53.2% variance in PM success, and PM success defines a 42.2% variance in overall project success. By addressing the identified success factors, the chances of success of public health projects increase. There is still 46.8% variance in PM success and 57.8% variance in overall project success that remain unexplained by these constructs and should be addressed in future research. This is not surprising, as previous work was unable to explain more than 45% of project success, and confirms the challenge of building appropriate constructs of overall project success^43^.

The research findings show that enhanced PM success and overall project success can be achieved by focusing on the success factors. The Spearman’s correlations and SEM results confirm that the following: “Resourcing” contributes to project success; “Communication and cohesion” contribute to project success; “Project team” highly contributes to project success; “Execution and control” highly contribute to project success; “PM success” highly contributes to overall project success.

### “Resourcing” contributes to PM success

Adequate resources (human, financial, material) should be allocated to the project and managed properly to increase the chances of success. The construct “Resourcing” comprises a realistic budget, a commitment to allocate funds, and ensuring that resources are available when necessary^74^. Oftentimes, success is only achieved if project benefits outweigh the costs and there is also a timely Return On the Investment (ROI). So, the business case, the cost-benefit, cost-effectiveness, or cost-utility analysis must be managed carefully during project implementation^76,78^. Having a realistic budget and a commitment to allocate funds represent a constraint for many healthcare organizations because there is a gap between their possibilities and the degree of ambition of their goals, so usually budgets are not adjusted to project goals^75,76,78,79,87^. Furthermore, strategy and decisions are influenced by politics since governmental decisions frequently change steering committees, so resource allocation is often compromised at any time during project implementation by the lack of medium/long-term commitments with top management. In many cases, “relaunching” the project – approaching it as a “new one” and with “new ownership” – is the only option for “regaining” support from top managers and ensuring that resources (human, physical, material, financial) are available when necessary. So, it is also important to ensure that the earned value of the project is controlled. If costs incurred for the work performed are higher than the planned costs, the causes must be identified, and adequate corrective measures should be implemented in a timely fashion^51^.

### “Communication and cohesion” contributes to PM success

Good personal internal and external communication abilities, as well as well-organized information and dynamic communication flow, increase the levels of success. Communication is more than a process of exchanging messages and implicates making them trustworthy, appropriate, relevant, and understandable by the audience^41^, as well as effective, efficient, and consistent for all project stakeholders^51^. When there is a lack of communication, any problem that arises may not be solved or may take a long time to be solved^21^. This result is consistent with previous research^74–76,79,82,87^. Trust and cohesion within the project team and between project stakeholders clearly contribute to project success^82^, as projects have gradually become temporary social networks of stakeholders that are committed to obtaining certain benefits^111^. Many interpersonal factors explain the quality of communication among project players, and this aspect influences success, both globally and at the level of some success criteria^82^. Therefore, the project manager should carefully manage informal communications and implement a formal communications plan to get everybody involved and committed to the project. The plan should consider: (1) the continuous technological evolution of society (new information and communication technologies, social networks); (2) increasing demands for digital access to information (by accessing digital clouds, for example); (3) increasing of virtual teams (people working, for example, in different countries); (4) results from dissemination in partnership with key stakeholders. Furthermore, every project plan should include a project start-up event and a project closure event^51,112^.

### “Project team” contributes to PM success

Organizing and managing a high-performing team strongly influences project success. In fact, project team commitment, motivation, and experience contribute to project success^14^. Organizing a multidisciplinary group, building confidence, trust, cohesion, and good interpersonal relationships between all team members positively impact teamwork and both individual and group performance. Focusing on these aspects should be actively promoted by the project manager. Project managers should also consider the characteristics of high-performance teams^21,100^: commitment, communication, empowerment, competence, cohesion and interdependence, diversity, structure, and recognition. A performance evaluation system may assess team performance through effectiveness and efficiency. High performance is intrinsically linked to motivation for carrying out the project^41,74–76,79,99,100^. The strategy of project-oriented organizations should not only focus on team performance during project implementation but also on developing and maintaining internal organizational capabilities regarding project management. This process depends on finding and maintaining good project managers and incorporating project management skills into their collective knowledge. Investment in project management training programs is a reality of many organizations when seen as a vehicle to identify and prepare good *in-house* project managers and capitalize on their performance regarding organizational development^76^. Organizations should also provide a skills development plan for workers, as well as effective internal professional development opportunities. The training programs must be tailored to resource needs and should address the development of relevant scientific and technical competencies in the scientific area of the project, as well as the development of project management skills^76^.

### “Execution and control” contributes to PM success

Project management success is intrinsically linked to the quality of project execution and control processes. This conclusion is supported by previous work^42,59,76–78,83,89,113^. Projects benefit from using specific project management tools and techniques (e.g., work breakdown structure, organizational breakdown structure, communication plan, risk matrix)^114^, from defining and implementing adequate logistics, establishing agile processes, the ability to manage the unforeseen and errors in a timely fashion and to overcome the resistance to change imposed by the project. Furthermore, every project plan should include short periodic meetings with the team, meetings with the steering committee and partners, and control reports on the project. Project management standards like ICB^51^ and PMBOK^112^ have been addressing the discussion on this subject.

### “PM success” highly contributes to overall project success

The results also show that performing well in project management is a good predictor of overall project success. As the average value for PM success is lower than the value for project success, we may argue that besides failing to finish on time, within budget, with the planned quality, and with satisfied stakeholders, many public health projects are seen as successful. Nevertheless, our results show that in public health projects, project management success strongly influences the overall project success, and these projects have important particularities that should be considered in their management^89^.

### Additional insights

Other interesting results emerged from this study. No statistically significant relationship was found between the success of public health projects and their strategic character for the organization, their political priority, their public interest, and the clarity of their mission, all success factors that fall within the construct “environment and mission”. A previous study supports this conclusion^43^ and justifies it by considering this explanatory construct as a macro-managed component of the organization’s governance that may not be visible at the operational level. It should also be noted that a significant proportion of the projects included in our sample were funded by a European funding program or by a private funding grant, so they are less vulnerable to environmental factors. We believe that the opportunism that characterizes many of the decisions regarding projects promoted by government-funded organizations is minimized in this context, an idea that is also supported by Dwyer et al.^76^. Likewise, public health projects usually involve the management of public stakeholders, the participation of the community, great involvement and participation of the informal and formal structures of the project context, addressing its power and interests, which are the success factors intrinsically linked to the construct “stakeholder management”. These action paradigms of public health may have less influence on projects sponsored by grants or private initiative. Therefore, it is necessary to test the robustness of this lack of effect in a sample of entirely publicly funded health projects.

Regarding the construct “organizational culture”, the respondents’ answers reveal cultural differences between permanent and temporary organizations that remain over time. The results support the thesis that projects are often “islands” (or “silos”) within organizations and have their own culture and dynamics, and so are not systematically influenced by the environment, culture, values and working patterns and habits of the permanent organization^115–118^, which is not necessarily a negative trait. It may be interesting to explore whether this is a risk or a protective factor in future research.

Regarding the “planning”, “project manager” and “monitoring and evaluation” constructs, we did not find arguments in the literature that can explain the absence of statistically significant individual relationships with the success achieved, so we believe that this corresponds to one or both of the following conditions: (1) the “planning” and “monitoring and evaluation” constructs are sometimes considered inherent to project management and therefore are not identified by the modeling procedure; (2) the meaningfulness of these explanatory constructs is hard to establish in a questionnaire. This conclusion justifies conducting additional research since it does not conform to generally accredited project management practices.

Concerning the response constructs, we identified that project impact on the target population was not a good predictor of the perceived overall project success. This is hardly surprising since 32% of the respondents revealed that it was not possible to assess the impact of the project on the target population’s health. This aspect should also be carefully addressed in future research because it represents a constraint in evaluating the effectiveness of public health projects and compromises investments in health promotion and prevention programs by public and private organizations. Many projects included in the sample have been completed recently, although we believe that this lack of evidence of the positive effects of the project on the target population is still a matter of concern.

## Conclusions

To the best of our knowledge, this is the first study that explores the manageable determinants of successful public health projects. We evidenced the strong relationship between certain conditions (success factors) and the success achieved in projects. The success factors should be considered for all the parties involved in project financing, planning, implementation, and evaluation, such as program and project owners, project managers, project teams, project officers, financing agencies, stakeholders, and top managers. This knowledge also has operational value for project management, as it can be used as a forecasting, diagnostic, and management tool by using the identified success factors as a checklist throughout the projects’ life cycle, from start-up to closing, particularly when addressing critical issues of project implementation. Therefore, the main recommendation of this study is that project-oriented organizations should take into account the proposed project management framework based on a model of success factors to enhance project success.

Another recommendation is that organizations should raise the awareness (e.g., by organizing training sessions) of project teams and stakeholders that all elements of the model are relevant for project success, even the less objective and subjective ones, such as “organizational culture”. Such elements should be clear and meaningful to employees, as they are the basis of some important project management competencies.

Our study has some limitations that can be addressed in future research. The responses were obtained from individuals (project managers and project team members) responsible for project implementation, so the survey data suffer from potential participant bias, which is a limitation also pointed out in other studies^43,119^. We believe that such bias was minimized by submitting an anonymous online questionnaire. In the future, extending the participation to other stakeholders — such as decision-makers, financiers, groups, or individuals served or affected by the project — can help clarify this issue. In addition to this potential bias, there are drawbacks to relying on memory in retrospective studies, as well as the challenge of recording facts about projects that have already been completed. To minimize this effect, we excluded projects finished more than 24 months before the date of invitation to participate. This seems to be an acceptable differential because it allows for the capture of medium- and long-term goals and results, not only those regarding the operational management of the project. Even though, it should be noted that, in some projects, their effects can come even later than 24 months. It should also be noted that most of the published studies are retrospective, so this option was taken according to the literature and is positive because it benefits from the maturity of initiatives^36,42,43,82,89,119,120^. We consider that future research should also focus on prospective studies that address the effects of dynamics between the project and its environment by including ongoing projects in different stages of implementation^115,121^.

Another suggestion for future research is the identification and characterization of project success factors throughout the project lifecycle, as was done by Hyväri^122^ and suggested by a variety of authors (e.g., Belout and Gauvreau^42^). It should be assessed whether factors that influence success at a tactical or planning stage differ from the major influencers in the operational implementation stage. This knowledge can provide important contributions to building a lifecycle management framework for public health projects.

This model can also be tested with other metrics and methods. In the future, we suggest determining the performance of project management using quantitative metrics of effectiveness and efficiency (including more explicit value-based metrics as proposed by Pereira et al.^52^ and Varajão and Trigo^123^), without neglecting the subjective nature of project management evaluation. This means that success can be assessed both by internal measures (e.g., objectives, budget and schedule, technical and business performance) and external measures (e.g., stakeholder satisfaction, value creation, effective target benefits, and future growth).

## Electronic supplementary material

Below is the link to the electronic supplementary material.

Supplementary Material 1

## Data Availability

The datasets generated and analyzed during the current study are not publicly available due to confidentiality reasons but are available (in anonymized form) from the corresponding author upon reasonable request.
