# Supplementary material for: Model of driving factors for success in public health project management using structural equation modeling
Source: Sci Rep. 2024 Oct 20;14:24647. doi: 10.1038/s41598-024-75437-7 (PMC11491464; doi:10.1038/s41598-024-75437-7)
Supplement: Supplementary file 1 — Supplementary Material 1 [file 41598_2024_75437_MOESM1_ESM.docx]

**Model of driving factors for success in public health project management using structural equation modeling**

Carolina Santos, João Varajão, Nilton Takagi, A. Manuela Gonçalves

# Appendix

| **Construct** | **SF code** | **Variable description** | **Scale** |
| --- | --- | --- | --- |
| Mission and environment | SF1 | The project is committed to a recognized priority in the field of public health | 7-Point Likert  (1- total  disagreement;  7- total  agreement) |
|  | SF2 | The project is strategic in the political, economic and social contexts |  |
|  | SF3 | The project has a public interest that is socially recognized |  |
|  | SF4 | The project is strategic in several governmental cycles |  |
|  | SF5 | The project is strategic for the promoting organization |  |
|  | SF6 | The project contributes to the achievement of goals defined in the National Health Plan and in Priority Health Programs |  |
|  | SF19 | Mission and vision are shared between the strategic partners |  |
|  | SF33 | The goals are clearly defined |  |
|  | SF49 | The team knows and shares project's goals, mission and vision |  |
|  | SF50 | The team is project-committed |  |
| Organizational culture | SF9 | The promoting organization has a project-oriented culture | 7-Point Likert  (1- total  disagreement;  7- total  agreement) |
|  | SF10 | The promoting organization has a low level of bureaucracy |  |
|  | SF11 | The promoting organization has a good working environment |  |
|  | SF12 | The promoting organization works flexibly and creatively |  |
|  | SF13 | The promoting organization fosters continuous improvement |  |
|  | SF14 | The promoting organization incorporates progress, research and new knowledge in timely fashion |  |
| Stakeholder management | SF7 | Partnerships include public organizations | 7-Point Likert  (1- total  disagreement;  7- total  agreement) |
|  | SF8 | Regional and local structures are involved in the project |  |
|  | SF15 | The interests of the partners are properly managed |  |
|  | SF16 | The promoting organization has the ability to align the partners' positions by taking into consideration their interests in the project |  |
|  | SF17 | The promoting organization captures the trust of all involved regarding project results, regardless of sponsorships |  |
|  | SF18 | The strategic partners of the project are duly identified |  |
|  | SF20 | The strategic partners of the project are properly involved in project planning and implementation |  |
|  | SF21 | The community is involved and participates in the project |  |
|  | SF22 | Partnerships increase during project implementation (e.g., for scope extension and/or results dissemination) |  |
|  | SF23 | The planning promotes transversality: broad participation and involvement |  |
|  | SF68 | Stakeholders management is supported by a stakeholder management matrix |  |
|  | SF79 | Project management includes regular meetings with the end-users or project beneficiaries |  |
| Planning | SF24 | Project planning is carried out with accuracy and consistency | 7-Point Likert  (1- total  disagreement;  7- total  agreement) |
|  | SF25 | The planned actions express a deep knowledge of the target population |  |
|  | SF34 | Success criteria are clearly defined |  |
|  | SF35 | Success factors are clearly defined |  |
|  | SF36 | Methodologies defined for the completion of goals are appropriate |  |
|  | SF45 | Risks and opportunities of the project are identified |  |
|  | SF46 | Risks, actions and contingency plans are defined |  |
|  | SF69 | Scope is detailed in phases, work packages, activities, and milestones |  |
|  | SF72 | Quality processes are formally defined in a quality management plan |  |
|  | SF74 | Project planning features detailed timeline (Gantt chart ) |  |
| Resourcing | SF26 | Investments are sustainable in the long-term | 7-Point Likert  (1- total  disagreement;  7- total  agreement) |
|  | SF27 | Return of the investment takes place (overall benefits exceed costs) |  |
|  | SF28 | Budget is adjusted to project scope |  |
|  | SF29 | Budget includes a buffer for project execution |  |
|  | SF30 | Resources (human, financial, material) are available when necessary |  |
| Monitoring and evaluation | SF39 | The information system that supports the project has quality | 7-Point Likert  (1- Total  disagreement;  7- Total  agreement) |
|  | SF40 | Key performance indicators (KPI) of the project are defined |  |
|  | SF41 | Key performance indicators (KPI) of the project are monitored with appropriate frequency |  |
|  | SF59 | The project has a rewarding performance system |  |
| Communication and cohesion | SF37 | New information and communication technologies are incorporated (e.g., social networks) | 7-Point Likert  (1- Total  disagreement;  7- Total agreement) |
|  | SF42 | Information and communication flows are defined and well-organized |  |
|  | SF43 | Internal communications (within the project team) are effective |  |
|  | SF44 | External communications (outside the project team) are effective |  |
|  | SF47 | Results dissemination is supported by a plan |  |
|  | SF54 | Cohesion, trust, and cooperation within the project team is preserved |  |
|  | SF73 | The project has a communication plan that identifies formal internal and external communications |  |
|  | SF75 | Project start-up includes a kickoff event |  |
|  | SF80 | Project close-out includes a closing event |  |
| Project team | SF51 | Project team members are highly motivated | 7-Point Likert  (1- Total  disagreement;  7- Total  agreement) |
|  | SF58 | Project team members benefit from competence training programs tailored to their needs |  |
|  | SF60 | Project team members show high performance in their role |  |
|  | SF61 | Project team members have high technical and scientific competencies in the scientific area of the project |  |
|  | SF62 | Project team members have high expertise in project management |  |
|  | SF65 | Project team members have high technical and scientific competencies in the scientific area of the project |  |
|  | SF66 | Team organization ensures multidisciplinarity |  |
| Project manager | SF52 | Project team members are aware of the boundaries of project work and their responsibilities | 7-Point Likert  (1- Total  disagreement;  7- Total  agreement) |
|  | SF53 | The expectations of project team members are properly managed |  |
|  | SF55 | The management of conflicts within the project team is effective |  |
|  | SF57 | The performance of the team and of each individual member is recognized |  |
|  | SF63 | The project manager has high skills in project management |  |
|  | SF64 | The project manager has high experience in project management |  |
| Execution and control | SF31 | Unexpected events and errors are managed properly | 7-Point Likert  (1- Total  disagreement;  7- Total  agreement) |
|  | SF32 | Critical incidents are solved in timely fashion |  |
|  | SF38 | The logistics for project implementation is ensured |  |
|  | SF48 | Resistance to change imposed by the project is overcome |  |
|  | SF67 | Project planning and implementation follow already tested project management methodologies (e.g., PMBOK) |  |
|  | SF70 | Hierarchical relationships and reporting processes within the project team are formally defined |  |
|  | SF71 | The responsibilities of each team member are formally defined in a responsibilities matrix |  |
|  | SF76 | Project status reports are produced periodically |  |
|  | SF77 | Project team meetings are organized periodically |  |
|  | SF78 | Project steering meetings (with the project owner and stakeholders) are organized periodically |  |
| PM success | SC1 | Goals (in the short, medium and long-term) | Ordinal (1- not assessed; 2- not achieved; 3- under planned; 4- as planned; 5- above planned) |
|  | SC2 | Scope (work) | Ordinal (1- not assessed; 2 - not done; 3 - under planned; 4 - as planned, with changes; 5 - as planned; 6 - above planned) |
|  | SC3 | Time (schedule) | Ordinal  (1-  abandoned;  2 - finished with delay; 3 - finished within schedule, with changes; 4- finished within schedule; 5 - finished in advance) |
|  | SC4 | Costs (budget) | Ordinal (1 - not assessed; 2 - over budget; 3 - within budget, with changes;  4 - within budget; 5 - under budget) |
|  | SC5 | End-users satisfaction | Ordinal (1 - not assessed; 2 - below expectations;  3- within  expectations; 4 - exceeding expectations) |
| Overall Project success | SC6 | Global success score | Ordinal (1 - total failure; 7 - total success) |

Table 5. Constructs, variables, scale

The explanatory constructs comprise 79 success factors, all measured by a 7-point Likert scale (1 - Totally disagree; 7 - Totally agree): environment and mission (10 items: SF1, SF2, SF3, SF4, SF5, SF6, SF19, SF33, SF49, SF50); organizational culture (6 items: SF9, SF10, SF11, SF12, SF13, SF14); stakeholder management (12 items: SF7, SF8, SF15, SF16, SF17, SF18, SF20, SF21, SF22, SF23, SF68, SF79); planning (10 items: SF24, SF25, SF34, SF35, SF36, SF45, SF46, SF69, SF72, SF74); resourcing (5 items: SF26, SF27, SF28, SF29, SF30); monitoring and evaluation (4 items: SF39, SF40, SF41, SF59); communication and cohesion (9 items: SF37, SF42, SF43, SF44, SF47, SF54, SF73, SF75, SF80); project team (7 items: SF51, SF58, SF60, SF61, SF62, SF65, SF66); project manager (6 items: SF52, SF53, SF55, SF57, SF63, SF64); and execution and control (10 items: SF31, SF32, SF38, SF48, SF67, SF70, SF71, SF76, SF77, SF78).

The responsive constructs comprise six success criteria: project goals (5-point ordinal scale); project scope (6-point ordinal scale); project schedule (5-point ordinal scale); project budget (5-point ordinal scale); end-user satisfaction (4-point ordinal scale); and global success score (7-point ordinal scale).

After confirming factorial analysis, five variables (SF33, SF49, SF74, SF52, and SF55) were removed, and 19 correlations were made between the errors of the items: SF56 and FS57; SF34 and SF35; SF2 and FS4; SF76 and FS77; SF70 and SF71; SF18 and SF20; SF31 and SS32; SF46 and SF72; SF7 and SF8; SF9 and SF10; SF3 and SF19; SF18 and SF21; SF69 and SF72; SF46 and SF45; SF73 and SF75; SF48 and SF77; SF60 and SF61; SF4 and SF5; SF28 and SF29.

The data were analyzed to detect outliers by using the protocol described by Tabachnick and Fidell [124], which classifies multivariate outliers as observations in which the Mahalanobis Distance is higher than the value of χ2 (85) = 131.041. This process revealed that there are no multivariate outliers.
